# The oxysterol and cholestenoic acid profile of mouse cerebrospinal fluid

**DOI:** 10.1016/j.steroids.2015.02.021

**Published:** 2015-07

**Authors:** Peter J. Crick, Lien Beckers, Myriam Baes, Paul P. Van Veldhoven, Yuqin Wang, William J. Griffiths

**Affiliations:** a College of Medicine, Grove Building, Swansea University, Singleton Park, Swansea SA2 8PP, UK; b Department of Pharmaceutical and Pharmacological Sciences, Laboratory of Cell Metabolism, Campus Gasthuisberg O/N2, B 3000 Leuven, Belgium; c Department of Cellular and Molecular Medicine, LIPIT, Campus Gasthuisberg O&N1, B3000 Leuven, Belgium

**Keywords:** CSF, cerebrospinal fluid, CYP, cytochrome P450, LXRs, liver X receptors, 7α,25-diHC, 7α,25-dihydroxycholesterol (cholest-5-ene-3β,7α,25-triol), 26-HC, (25R)26-hydroxcholesterol,(cholest-5-en-3β,(25R)26-diol), BBB, blood brain barrier, CNS, central nervous system, EADSA, enzyme assisted derivatisation for sterol analysis, LC–MS^n^, liquid chromatography–tandem mass spectrometry, 22R-HCO, 22R-hydroxycholest-4-en-3-one, GP, Girard P, SPE, solid phase extraction, LIT, linear ion trap, RIC, reconstructed ion chromatograms, 7-OC, 7-oxocholesterol (3β-hydroxycholest-5-en-7-one), 3β-HCA, 3β-hydroxycholest-5-enoic acid, 3β,7α-diHCA, 3β,7α-dihydroxycholest-5-enoic acid, 7αH,3O-CA, 7α-hydroxy-3-oxocholest-4-enoic acid, 7α,24-diHCO, 7α,24-dihydroxycholest-4-en-3-one, LC–MS, Derivatisation, Bile acid, Brain

## Abstract

Oxysterols and cholestenoic acids are oxidised forms of cholesterol with a host of biological functions. The possible roles of oxysterols in various neurological diseases makes the analysis of these metabolites in the central nervous system of particular interest. Here, we report the identification and quantification of a panel of twelve sterols in mouse cerebrospinal fluid (CSF) using liquid chromatography–mass spectrometry exploiting enzyme assisted derivatisation for sterol analysis technology. We found low levels of oxysterols and cholestenoic acids in CSF in the range of 5 pg/mL–2.6 ng/mL. As found in man, these concentrations are one to two orders of magnitude lower than in plasma.

## Highlights

• Measurement of oxysterols and cholestenoic acids in mouse CSF by LC–MS.
• Major cholesterol metabolites in mouse CSF are 7α-hydroxylated cholestenoic acids.
• Levels of cholesterol metabolites an order of magnitude lower in CSF than plasma.
• 7α-Hydroxy metabolites of 24S-hydroxycholesterol found in CSF but not plasma.

## Graphical abstract

**Figure:**
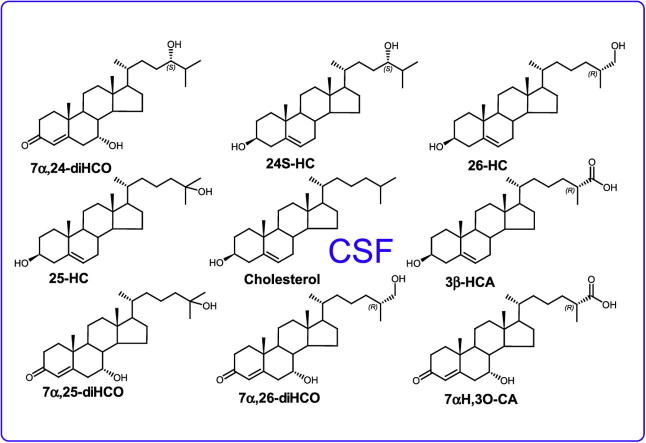

## 1 Introduction

Oxysterols and cholestenoic acids are formed from cholesterol as intermediates in the synthesis of bile acids and steroid hormones. Oxidation of cholesterol is catalysed by numerous sterol hydroxylases, primarily members of the cytochrome P450 (CYP) family of enzymes [1]. Although the presence of oxysterols in mammalian circulation has been known for many years, recent studies have led to a renewed interest in these metabolites as they have been shown to possess potent biological activities. For example, 24S,25-epoxycholesterol is a ligand to the liver X receptors (LXRs) [2], 7α,25-dihydroxcholesterol (7α,25-diHC) plays a role in immune cell migration by binding to the Epstein-Barr virus induced gene 2 (EBI2) [3,4], and (25R)26-hydroxycholesterol (26-HC) has been shown to influence tumour growth by modulating the estrogen receptor β (ERβ) [5,6]. Note, here we use the systematic nomenclature where addition of a hydroxy group on the terminal carbon of the cholesterol side-chain introducing R stereochemistry at C-25, is at C-26 giving (25R)26-hydroxycholesterol. The common, but systematically incorrect, name for this molecule is 27-hydroxycholesterol [7,8].

The high level of cholesterol in the brain (approximately 2% wet weight of brain) has stimulated research investigating altered cholesterol metabolism as a causative factor in neurological diseases. For example, oxysterol levels have been shown to be perturbed in neurodegenerative diseases including Alzheimer’s disease [9] and Parkinson’s disease [10], while altered metabolism of 25-HC has been linked to neuroinflammation in mouse models [11]. Recently, it has been demonstrated that oxysterols promote midbrain neurogenesis [12] while cholestenoic acids regulate the survival of motor neurons both in *vitro* and in *vivo* [13].

Mouse models of neurological diseases are an important resource to investigate the causes and treatments of these conditions. Increasingly sophisticated mouse models for numerous diseases are becoming available, as well as knockout animals with mutations of specific enzymes involved in sterol homeostasis. To link observed biological phenomena to the underlying molecular causes it is useful to analyse the levels of oxysterols and cholestenoic acids in different tissues of the mouse. Blood plasma is often used for analysis as it is straightforward to collect and gives a “snapshot” of oxysterol levels throughout the body. However, the presence of the blood brain barrier (BBB) raises questions of the relevance of plasma measurements in the context of neurological diseases. Previously we have analysed brain tissue which circumvents this problem and allows direct analysis of metabolites that may play a role in disease [14]. While this is a powerful approach, translation to human studies is only possible for post mortem analysis.

An alternative is to measure oxysterols in cerebrospinal fluid (CSF). This has the advantage of relatively straightforward sample collection in humans while representing conditions in the central nervous system (CNS) more faithfully than blood plasma. We have previously reported the identification and quantitation of about 25 oxysterols and cholestenoic acids in human CSF [15] and shown that inborn errors of cholesterol metabolism affect the levels of the metabolites present [13]. However, to the best of our knowledge, there have been no reports of the oxysterol profile of mouse CSF which makes comparisons between human diseases and mouse models difficult.

Here, we present a robust method for the identification and quantification of cholesterol metabolites in mouse CSF that opens new possibilities for biomarker discovery and investigations into the underlying causes of neurological diseases. We compare this data with concentrations of oxysterols and cholestenoic acids found in plasma to give an overview of cholesterol metabolism in mouse.

## 2 Experimental

### 2.1 Sample collection

CSF was from male (∼40 g) and female (∼34 g) Swiss/Webster mice (4–6 months), sedated with an overdose of Nembutal (150 μg/g body weight). CSF was collected from the cisterna magna as described by Liu and Duff [16] but only a single sampling of 5–10 μL was performed and the mouse was subsequently sacrificed. Care was taken to avoid blood vessels when penetrating the dura mater. Pools of 100 μL CSF for male mice and 54 μL CSF for female mice were made. Mice were bred in the animal housing facility of the KU Leuven, had ad libitum access to water and standard rodent food, and were kept on a 12 h light and dark cycle. All animal experiments were performed in accordance with the “Guidelines for Care and Use of Experimental Animals” and fully approved by the Research Advisory Committee (Research Ethical committee) of the KU Leuven. Male mouse (B6, 3 months of age) plasma was purchased from Jackson Laboratories (Maine, USA).

### 2.2 Analysis of oxysterols and cholestenoic acids

Oxysterols and cholestenoic acids from mouse plasma were analysed using enzyme assisted derivatisation for sterol analysis (EADSA) and liquid chromatography–tandem mass spectrometry (LC–MS^n^) by the method previously described [17]. Metabolites from mouse CSF were analysed using a similar method, but with the modifications described below.

### 2.3 Extraction of oxysterols and cholestenoic acids from mouse CSF

CSF (54–100 μL) was added to ethanol (1.05 mL, Fisher Scientific) containing 24R/S-[25,26,26,26,27,27,27-^2^H_7_]hydroxycholesterol ([^2^H_7_]24R/S-HC) (2 ng), 22R-[25,26,26,26,27,27,27-^2^H_7_]hydroxycholest-4-en-3-one ([^2^H_7_]22R-HCO) (2 ng), 7α,25-[26,26,26,27,27,27-^2^H_6_]dihydroxycholesterol ([^2^H_6_]7α,25-diHC) (0.2 ng) and [25,26,26,26,27,27,27-^2^H_7_]cholesterol (20 μg) (all Avanti Polar Lipids) in an ultrasound bath. After 5 min water (Fisher Scientific) was added to give a final volume of 1.5 mL of 70% ethanol. The mixture was centrifuged for 30 min at 17,089×*g* at 4 °C. To remove cholesterol from the sample a solid phase extraction (SPE) step was used. A 200 mg tC_18_ Sep-Pak cartridge (Waters) was preconditioned with ethanol (4 mL) followed by 70% ethanol (6 mL) after which the sample was loaded. The flow-through was combined with a wash of 70% ethanol (5.5 mL) to give a 7 mL fraction containing oxysterols and cholestenoic acids. The solvent was evaporated in a vacuum centrifuge overnight.

### 2.4 Enzyme assisted derivatisation of oxysterols and cholestenoic acids

The dried sample was dissolved in propan-2-ol (50 μL, Fisher Scientific) and 50 mM KH_2_PO_4_ buffer (500 μL) then treated with 3 μL of cholesterol oxidase (2 mg/mL in H_2_O, 44 units/mg protein, Sigma–Aldrich) and incubated for 1 h at 37 °C. The reaction was quenched by addition of methanol (1 mL) followed by glacial acetic acid (75 μL, VWR) and Girard P (GP, TCI Europe) reagent (75 mg). After vortexing, the mixture was incubated at room temperature in the dark overnight.

Excess reagent was removed by SPE using a 50 mg tC_18_ Sep-Pak cartridge (Waters) preconditioned with methanol (1.5 mL, Fisher Scientific), 10% methanol (1.5 mL) and 70% methanol (1 mL). The sample was loaded and allowed to flow through the cartridge. To ensure full recovery of all analytes of interest, a recycling procedure was used where the eluate was diluted with an equal volume of water and reapplied to the column. This procedure was repeated to give a final concentration of 17% methanol. The cartridge was then washed with 10% methanol (1.5 mL) and the analytes of interest eluted with methanol (3 × 250 μL to give Fr-1, Fr-2 and Fr-3) followed by ethanol (250 μL to give Fr-4). The solvent was evaporated from combined Fr-1 and Fr-2 using a vacuum centrifuge and the sample was re-suspended in 60% methanol (100 μL) for analysis by LC–MS^n^.

### 2.5 LC–MS^n^ on the LTQ-Orbitrap

Oxysterols were separated using a RSLC nano Ultimate 3000 (Dionex) with a Hypersil Gold C_18_ column (1.9 μm particle size, 50 × 2.1 mm, Thermo Fisher). Mobile phase A consisted of 33.3% methanol, 16.7% acetonitrile (Fisher Scientific), 50% water, containing 0.1% formic acid (VWR) while mobile phase B was 63.3% methanol, 31.7% acetonitrile, 5% water, containing 0.1% formic acid. The gradient started at 20% mobile phase B for 1 min before increasing to 80% mobile phase B over 7 min. After holding for 5 min the gradient returned to 20% B over 6 s before re-equilibration for 3 min 54 s to give a total run time of 17 min. The flow rate was 200 μL/min and the eluent was directed to the atmospheric pressure ionisation source of an LTQ-Orbitrap Velos (Thermo Fisher). Eighty five microlitre of the derivatised mouse CSF was injected and a full scan was performed in the Orbitrap across the *m*/*z* range 400–610 at 30,000 resolution (full width at half maximum height). At the same time the linear ion trap (LIT) monitored MS^n^ transitions for GP tagged oxysterols and cholestenoic acids. Initial activation gave a characteristic [M-79]^+^ fragment in MS^2^ corresponding to the loss of pyridine, the [M-79]^+^ ion was isolated and when activated further gave structurally informative MS^3^ spectra.

## 3 Results and discussion

### 3.1 Analysis using EADSA reveals 12 oxysterols and cholestenoic acids in mouse CSF

**Fig. 1:**
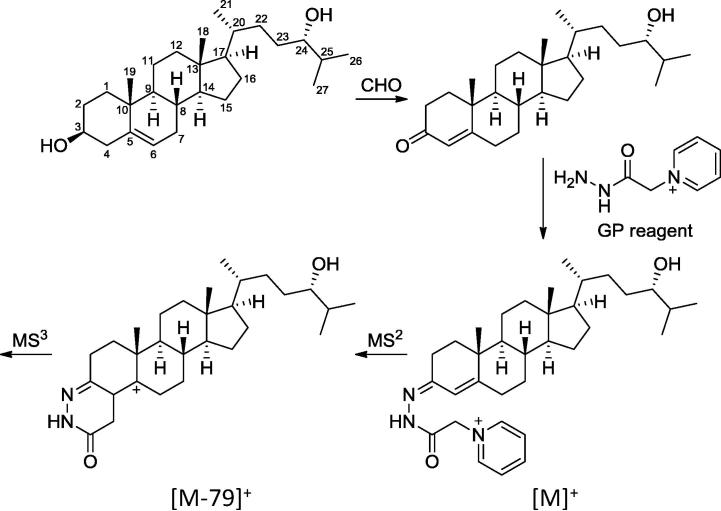
Numbering of the cholesterol backbone and outline of the EADSA strategy exemplified by 24S-HC. CHO: cholesterol oxidase.

We used EADSA to charge-tag sterols of interest with the GP reagent (Fig. 1). Enzymatic oxidation of the characteristic sterol 3β-hydroxy group is followed by derivatisation with the GP reagent which introduces a permanent positive charge to the analyte of interest in the form of a quaternary ammonium ion. This greatly enhances analyte signal allowing the detection of low levels of oxysterols in biological matrices. By including isotopically labelled internal standards quantification can be carried out with high accuracy and reproducibility [17,18].

To account for the very low levels of oxysterols (pg/mL) in mouse CSF, as well as the small amount of fluid available (5–10 μL per animal), we used pooled CSF samples (6–8 mice) and made several modifications to our procedure usually employed for plasma analysis. We used a smaller SPE cartridge (50 mg of sorbent cf. 200 mg for plasma) for the removal of the excess derivatisation reagent. In addition, before analysis we concentrated samples in a vacuum centrifuge to maximize the amount of analyte injected on the LC column. We worked up 100 μL of CSF pooled from male mice, and 54 μL of CSF pooled from females and injected 85% of each in a single injection.

**Fig. 2:**
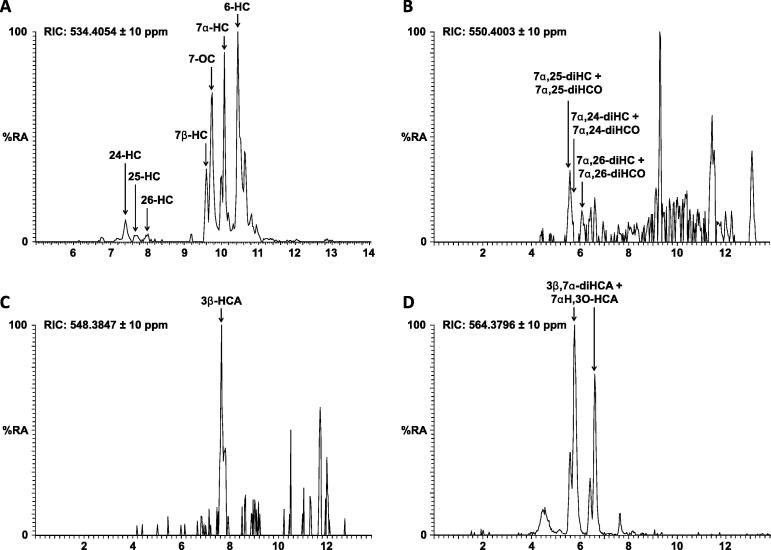
Oxysterols and cholestenoic acids in mouse CSF. (A) RIC for *m*/*z* 534.4054 showing 24S-HC, 25-HC, 26-HC and B-ring oxidised sterols. (B) RIC for *m*/*z* 550.4003 showing 7α,25-diHC/7α,25-diHCO, 7α,24-diHC/7α,24-diHCO, and 7α,26-diHC/7α,26-diHCO. (C) RIC for *m*/*z* 548.3847 showing 3β-HCA. (D) RIC for *m*/*z* 564.3796 showing 3β,7α-diHCA/7αH,3O-CA. Note that sterols derivatised with the GP reagent form a mixture of *syn* and *anti* isomers which may or may not be resolved e.g. 3β,7α-diHCA/7αH,3O-CA elute as *syn* and *anti* isomers at 5.77 and 6.61 min, and the second isomer of 7α,24-diHC/7α,24-diHCO elutes at 6.61 min.

Using the LTQ-Orbitrap Velos we recorded high resolution accurate mass spectra with an *m*/*z* error of <5 ppm. By plotting reconstructed ion chromatograms (RICs) for *m*/*z* values corresponding to different oxysterols, we could detect multiple metabolites. For example, a RIC for *m*/*z* 534.4054 shows the side-chain monohydroxycholesterols 24S-HC, 25-HC and 26-HC along with the B-ring oxidised metabolites 7α-HC, 7β-HC, 7-oxocholesterol (7-OC) and 6-HC (Fig. 2). Similarly, a RIC for *m*/*z* 548.3847 shows 3β-hydroxycholest-5-enoic acid (3β-HCA, Fig. 2), while a RIC of *m*/*z* 564.3796 shows 3β,7α-dihydroxycholest-5-enoic acid (3β,7α-diHCA) plus 7α-hydroxy-3-oxocholest-4-enoic acid (7αH,3O-CA) (Fig. 2). Note, using the EADSA methodology the sterol 3β-hydroxy group is oxidised to a 3-oxo group prior to GP derivatisation (Fig. 1) and sterols naturally possessing a 3-oxo group are differentiated from those modified to contain one by repeating the derivatisation reaction, with a differentially isotopically labelled GP reagent, in the absence of prior oxidation. Due to low levels of analytes and the scarcity of mouse CSF there was insufficient material to allow a repeat analysis so the values reported are the sum of the 3β-hydroxy and 3-oxo forms. Only analytes with a 7α-hydroxy group are substrates for the endogenous hydroxysteroid dehydrogenase enzyme HSD3B7 which in mouse oxidises the 3β-hydroxy group to a 3-oxo group in *vivo*. Thus, only metabolites with a 7α-hydroxy group can exist in a 3β and 3-oxo form in *vivo* (Table 1). In addition to monohydroxycholesterols the dihydroxy metabolites 7α,24-diHC plus 7α,24-dihydroxycholest-4-en-3-one (7α,24-diHCO), 7α,25-diHC plus 7α,25-diHCO and 7α,26-diHC plus 7α,26-diHCO were also identified (Fig. 2).

**Fig. 3:**
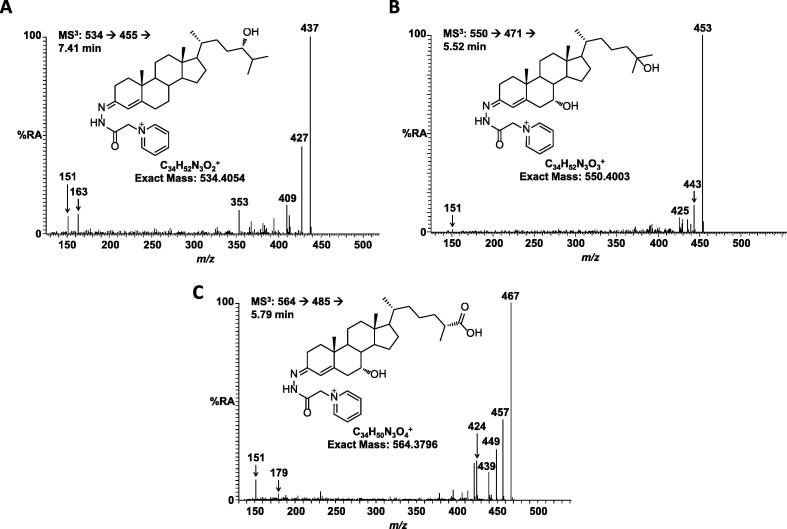
MS^3^ fragmentation patterns for oxysterols and cholestenoic acids in mouse CSF. (A) MS^3^ spectrum for the transition 534.4 → 455.4 → recorded at 7.41 min showing 24S-HC. Note the characteristic fragment at *m*/*z* 353.4. (B) MS^3^ spectrum for the transition 550.4 → 471.4 → recorded at 5.52 min showing 7α,25-diHC/7α,25-diHCO. (C) MS^3^ spectrum for the transition 564.4 → 485.4 → recorded at 5.79 min showing 3β,7α-diHCA/7αH,3O-CA.

Further confirmation of the identity of the metabolites was provided by MS^n^ spectra recorded in the LIT of the LTQ-Orbitrap. Fragmentation of sterols tagged with the GP reagent gives a characteristic peak at [M-79]^+^ corresponding to the loss of pyridine (Fig. 1). Isolation of this fragment and further activation (i.e. MS^3^) then gives structurally informative fragments unique to each individual metabolite. For example, Fig. 3A shows the MS^3^ spectrum of 24S-HC with fragments characteristic of a GP derivatised sterol, without a 7-hydroxy group present, at *m*/*z* 151.1, 163.1 and 177.1 along with compound specific fragments such as the distinctive peak at *m*/*z* 353.4. The major peak at *m*/*z* 437 corresponds to [M-79-H_2_O]. Other oxysterols and cholestenoic acids give different fragmentation patterns that can be compared with authentic standards to confirm the identity of each metabolite (Fig. 3).

By basing identification on LC retention time, accurate mass, and unique fragmentation patterns, we were able to detect 12 oxysterols and cholestenoic acids in mouse CSF (Table 1).

### 3.2 CSF concentrations of oxysterols are low in CSF in comparison to plasma, but 7α,24-diHC plus 7α,24-diHCO is only found in CSF

By comparison of the peak areas from RICs for each metabolite with those of deuterated internal standards we were able to quantify oxysterols and cholestenoic acids in mouse CSF. For comparison, we also analysed the oxysterol content of mouse plasma using our previously reported method. The levels of oxysterols and cholestenoic acids in mouse CSF are in the range of 5 pg/mL–2.6 ng/mL (Table 1). These are low concentrations in the context of levels present in mouse plasma where the range is from 0.4 to 28 ng/mL. In general, the level of each sterol is one to two orders of magnitude higher in plasma than CSF. The most abundant metabolites in both matrices are 3β,7α-diHCA plus 7αH,3O-CA at a concentration of about 2.5 ng/mL in CSF and 28 ng/mL in plasma. There is now a considerable body of evidence to indicate that 7αH,3O-CA and probably its precursor 3β,7α-diHCA are synthesised in brain from 26-HC, and that brain represents the likely source of these acids in CSF [13,15,19,20]. The origin of the acids in plasma is also likely to be extrahepatic and to represent a mode of reverse cholesterol transport to liver [21]. 7α,24-diHC plus 7α,24-diHCO is identified in CSF but not in plasma. 7α,24-diHC can be formed from 24S-HC through 7α-hydroxylation by the enzyme CYP39A1, however, it is not known if CYP39A1 is expressed in brain [1]. Oxysterol 7α-hydroxylase (CYP7B1) is known to be expressed in brain [1] and may have some activity towards 24S-HC, thus this enzyme could also be responsible for 7α-hydroxylation of 24S-HC in CNS. 24S-HC is synthesised in brain and is present at high levels (15–50 ng/mg) [15]. There are only small differences in the CSF oxysterol concentrations between male and female mice and it is unknown how the patterns of these important molecules change with age and with mouse strain. This may be the subject of future studies as it is known that the pattern of oxysterols in the newborn mouse is different from that of the adult [14].

A point to consider when performing CSF analysis is the possible contamination of the sample by blood during CSF collection. If CSF was contaminated by blood the expected ratio of 26-HC to 24S-HC in plasma and CSF would be similar. However the ratios are quite different, the plasma ratio of 26-HC to 24S-HC is about 3/2, while in CSF it is about 1/3. This data would argue against appreciable contamination of CSF with blood.

We have previously reported the detailed oxysterol and cholestenoic acid profiles of human plasma and CSF [13,15]. The levels of oxysterols and cholestenoic acids in human CSF are in some cases 5–10 times higher than in mouse, with a range of 26 pg/mL–25 ng/mL in human [13]. Similarly, the concentrations found in human plasma are higher than in mouse ranging from about 1 to 100 ng/mL in human.

## 4 Conclusions

LC–MS with EADSA technology allows detection and quantification of the extremely low levels of sterols found in mouse CSF which are about 1–2 orders of magnitude lower than in mouse plasma. A similar large difference is seen in man, reflecting the limited transport through the BBB. Also for other protein-bound lipids (e.g. long chain fatty acids) [22], such differences between serum and CSF levels have been described. These finding have to be considered when studying the effect of steroids on neuronal cells. The analysis of oxysterols and cholestenoic acids in mouse CSF offers new opportunities for biomarker discovery suitable for translation to human studies.

## Figures and Tables

**Table 1:** Oxysterols and cholestenoic acids identified and quantified in mouse CSF and plasma using EADSA. Mean plasma values are from 6 male B6 mice. Mean CSF values are for pooled samples from 6 to 8 Swiss/Webster mice.

After EADSA			Originating structure	CSF Male ng/mL (nmol/L)	CSF Female ng/mL (nmol/L)	Plasma ng/mL (nmol/L)
[M]^+^*m*/*z*	Formula	Sterol systematic name	Sterol systematic name (common name)			
534.4054	C_34_H_52_N_3_O_2_^+^	24S-Hydroxycholest-4-en-3-one 3-GP	Cholest-5-ene-3β,24S-diol (24S-hydroxycholesterol)	0.093 (0.230)	0.092 (0.229)	5.834 (14.500)
534.4054	C_34_H_52_N_3_O_2_^+^	25-Hydroxycholest-4-en-3-one 3-GP	Cholest-5-ene-3β,25-diol (25-hydroxycholesterol)	0.005 (0.012)	N.D.	1.216 (3.022)
534.4054	C_34_H_52_N_3_O_2_^+^	26-Hydroxycholest-4-en-3-one 3-GP	Cholest-5-ene-3β,26-diol ((25R)26-Hydroxycholesterol)	0.029 (0.073)	N.D.	8.364 (20.788)
534.4054	C_34_H_52_N_3_O_2_^+^	7b-Hydroxycholest-4-en-3-one 3-GP	Cholest-5-ene-3β,7β-diol (7β-Hydroxycholesterol)	0.137 (0.340)	N.D.	1.364 (3.390)
534.4054	C_34_H_52_N_3_O_2_^+^	3β-Hydroxycholest-5-en-7-one 7-GP	3β-Hydroxycholest-5-en-7-one (7-Oxocholesterol)	0.449 (1.116)	1.063 (2.642)	2.450 (6.089)
534.4054	C_34_H_52_N_3_O_2_^+^	7α-Hydroxycholest-4-en-3-one 3-GP	7α-Hydroxycholest-4-en-3-one or Cholest-5-ene-3β,7α-diol (7α-Hydroxycholesterol)	0.347 (0.862)	0.182 (0.452)	25.036 (62.224)
534.4054	C_34_H_52_N_3_O_2_^+^	6-Hydroxycholest-4-en-3-one 3-GP	Cholest-4-ene-3β,6-diol or Cholest-5-ene-3β,6-diol (6-Hydroxycholesterol)	0.677 (1.683)	0.638 (1.586)	1.466 (3.643)
548.3847	C_34_H_50_N_3_O_3_^+^	3-Oxocholest-4-en-26-oic acid 3-GP	3β-Hydroxycholest-5-en-26-oic acid	0.149 (0.357)	0.228 (0.548)	4.702 (11.294)
550.4003	C_34_H_52_N_3_O_3_^+^	7α,24-Dihydroxycholest-4-en-3-one 3-GP	7α,24-Dihydroxycholest-4-en-3-one or Cholest-5-ene-3β,7α,24-triol	0.056 (0.134)	0.028 (0.067)	N.D.
550.4003	C_34_H_52_N_3_O_3_^+^	7α,25-Dihydroxycholest-4-en-3-one 3-GP	7α,25-Dihydroxycholest-4-en-3-one or Cholest-5-ene-3β,7α,25-triol	0.044 (0.105)	0.033 (0.079)	0.491 (1.174)
550.4003	C_34_H_52_N_3_O_3_^+^	7α,26-Dihydroxycholest-4-en-3-one 3-GP	7α,26-Dihydroxycholest-4-en-3-one or Cholest-5-ene-3β,7α,26-triol	0.012 (0.028)	0.010 (0.025)	0.438 (1.047)
564.3796	C_34_H_50_N_3_O_4_^+^	7α-Hydroxy-3-oxocholest-4-en-26-oic acid 3-GP	7α-Hydroxy-3-oxocholest-4-en-26-oic acid or 3β,7α-Dihydroxycholest-5-en-26-oic acid	2.465 (5.703)	2.560 (5.921)	28.068 (83.687)
